# Factors contributing to sepsis-associated encephalopathy: a comprehensive systematic review and meta-analysis

**DOI:** 10.3389/fmed.2024.1379019

**Published:** 2024-05-21

**Authors:** Zhiyang Zhang, Li Guo, Lijing Jia, Hong Duo, Limin Shen, Heling Zhao

**Affiliations:** ^1^Department of Critical Care Medicine, Hebei Medical University, Shijiazhuang, China; ^2^Department of Intensive Care Unit, Hebei General Hospital, Shijiazhuang, China; ^3^Department of Neonatal, Shijiazhuang Fourth Hospital, Shijiazhuang, China; ^4^Zhongnan Hospital of Wuhan University, Wuhan, China

**Keywords:** sepsis, encephalopathy, delirium, risk factors, meta-analysis

## Abstract

**Background:**

This study aims to systematically assess the risk factors, the overall strength of association, and evidence quality related to sepsis-associated encephalopathy.

**Methods:**

A systematic search was conducted in the Cochrane Library, PubMed, Web of Science, and Embase for cohort or case-control studies published up to August 2023 on risk factors associated with sepsis-related encephalopathy. The selected studies were screened, data were extracted, and the quality was evaluated using the Newcastle–Ottawa Scale. Meta-analysis was performed using RevMan 5.3 software. The certainty of the evidence was assessed using the GRADE criteria.

**Results:**

A total of 13 studies involving 1,906 participants were included in the analysis. Among these studies, 12 were of high quality, and one was of moderate quality. Our meta-analysis identified six risk factors significantly associated with Serious Adverse Events (SAE). These included APACHE II, SOFA, age, tau protein, and IL-6, which were found to be risk factors with significant effects (standard mean difference SMD: 1.24–2.30), and albumin, which was a risk factor with moderate effects (SMD: −0.55). However, the certainty of evidence for the risk factors identified in this meta-analysis ranged from low to medium.

**Conclusion:**

This systematic review and meta-analysis identified several risk factors with moderate to significant effects. APACHE II, SOFA, age, tau protein, IL-6, and albumin were associated with sepsis-related encephalopathy and were supported by medium- to high-quality evidence. These findings provide healthcare professionals with an evidence-based foundation for managing and treating hospitalized adult patients with sepsis-related encephalopathy.

## Introduction

Sepsis is a life-threatening disease caused by a dysregulation of the body’s response to infection (1). To distinguish sepsis-induced brain dysfunction from intracranial infection, sepsis-associated encephalopathy (SAE) was introduced and defined as diffuse brain dysfunction caused by a systemic immune-inflammatory response to a disease without clinical or laboratory evidence of direct brain infection (2). Sepsis-associated encephalopathy (SAE) and sepsis-associated delirium (SAD) are closely related neurological manifestations of sepsis. SAD, a subtype of SAE, explicitly denotes the acute and fluctuating cognitive impairments that occur in the context of sepsis. While all patients with SAD have SAE, not all SAE patients exhibit the characteristic confusion and reduced awareness seen in delirium. SAE usually presents as an acute deterioration in mental status, manifested by cognitive confusion, impaired consciousness, disorientation, agitation, rigidity, and coma (3). The pathophysiologic basis of SAE is very complex and involves multiple mechanisms that lead to brain dysfunction and injury (4). One of its main mechanisms is the release of pro-inflammatory cytokines, which leads to the breakdown of the blood-brain barrier (BBB), causing the influx of immune cells and inflammatory mediators into the brain (5).

SAE is considered the most common cause of encephalopathy in the intensive care unit (ICU) (6). Sepsis has a variety of complications, among which sepsis-associated encephalopathy (SAE) is one of the critical clinical manifestations, and severe SAE can occur in about 30 to 70% of patients with sepsis (7). In a landmark study of 50 non-sedated ICU sepsis patients, SAE was observed in 54% of patients (8). The incidence reported in a specific French multicenter cohort of ICUs was 53 percent (9). SAE prevalence is up to 68% in US MIMIC-IV and eICU databases (10). The diagnostic criteria for sepsis have evolved from Sepsis 1.0 to Sepsis 3.0 (11–13). There are no standardized criteria for the diagnosis of SAE, and the following diagnostic criteria are currently in use: (1) cognitive and neuropsychiatric disorders documented by healthcare professionals (doctors and nurses); (2) manifestations of Delirium (diagnosed by assessment methods such as CAM-ICU); and (3) Glasgow Coma Score (GCS) <15 (6, 14–16). Numerous studies have shown that SAEs are associated with increased short-term mortality, prolonged hospitalization, or over-expenditure of healthcare resources and have the potential to cause permanent neurological sequelae (17–19).

This systematic review aimed to identify key risk factors for sepsis-associated encephalopathy. Historically, there have been several problems with research reviews on SAE, such as confusing diagnoses of SAE, different study sample sizes, and different clinical populations. Identifying risk factors for SAE will help develop and implement prevention strategies for patients with sepsis.

## Methods

### Study protocol

It is a systematic review and meta-analysis assessing risk factors for sepsis-associated encephalopathy. Before the study started, a proposal was developed and registered in PROSPERO (CRD42023483721) (see Supplementary Table S1 for PROSPERO protocol). This study followed the Preferred Reporting Items for Systematic Reviews and Meta-Analysis guidelines (20).

### Search strategy

We based search terms on Medical Subject Headings (MeSH) and other standard (controlled vocabulary) terms. A concept-based approach was used, including terms related to “Sepsis,” “Encephalopathy,” “Delirium,” “Risk Factors,” and others. Supplementary Table S2 details the entire search strategy. Two reviewers (ZZ and LJ) independently searched the Web of Science, Cochrane Library, Embase, and PubMed databases from inception to August 10, 2023. Our research team then searched the references of relevant studies to identify possible additional eligible studies. We used Endnote documentation software as a literature screening tool. Our research team repeated this process until we found no new relevant papers. Any discrepancies or differences were resolved through discussion or consultation with a third reviewer to reach a consensus.

### Inclusion and exclusion criteria

#### Inclusion criteria

##### Study subjects

We included studies involving human subjects aged 18 and above.

##### Case number requirements

Each study must include at least five cases of sepsis-associated encephalopathy (SAE), ensuring a representative dataset and statistical robustness.

##### Diagnostic tools

Included studies must utilize industry-recognized diagnostic or assessment tools for SAE to ensure consistency and accuracy in case diagnosis and evaluation (6, 14–16).

##### Type of study

Only studies assessing risk factors for the onset of SAE were included. Specifically, we focused on cohort, case-control, and cross-sectional studies as these designs are suitable for identifying associations between risk factors and disease occurrence.

##### Publication requirements

Studies must be peer-reviewed, published in scientific journals, and provide full-text articles for comprehensive quality assessment and data extraction.

#### Exclusion criteria

##### Specific patient groups

We excluded studies involving intracranial infections as their pathology and treatment could significantly differ from those of standard SAE patients, potentially skewing the results.

##### Study design limitations

We excluded case reports and case series involving four or fewer patients due to their insufficient data volume to support broad scientific conclusions. Similarly, literature reviews were excluded as they often rely on secondary data, which may not provide original data or detailed methods.

##### Language limitations

Only studies published in English were included to ensure accurate understanding and analysis of the data by our research team.

### Study selection and data extraction

Data extraction was independently and systematically conducted by two reviewers (ZZ and LG) using a pre-specified data extraction form; any disputes were resolved through consensus or adjudicated by a third reviewer (HZ) until the disagreement was resolved. Data collection included study characteristics: first author, publication year, study design, total number of patients, age, gender, incidence of SAE, sepsis diagnostic criteria, and SAE diagnostic criteria. The baseline characteristics of the included studies are illustrated in Table 1.

**Table 1:** Characteristics of the 13 studies.

Study	County	Study design	Age (year)	Male/female	Total patients	Cases number	Controls number	SAE incidence (%)	Sepsis diagnostic criteria	SAE assessment tools/diagnostic criteria
Li et al. (2011)	China	Case-control	51.1 ± 27.1	164/120	284	107	177	37.68%	Sepsis2.0	Data recorded by medical staff
Zhang et al. (2012)	China	Case-control	51.5 ± 14.8	157/75	232	41	191	17.67%	Sepsis2.0	Data recorded by medical staff
Zhao et al. (2019)	China	Cohort	61.7 ± 13.0	64/45	109	27	82	24.77%	Sepsis1.0	Data recorded by medical staff
Chen et al. (2023)	China	Cohort	64.3 ± 15.5	61/29	90	57	33	63.33%	Sepsis3.0	GCS <15
Lu et al. (2016)	China	Cohort	58.7 ± 8.4	57/29	86	34	52	39.53%	Sepsis2.0	Data recorded by medical staff
Kristo et al. (2018)	Finland	Case-control	64.2 ± 16.9	14/8	22	10	12	45.45%	Sepsis1.0	CAM-ICU
Chen et al. (2020)	China	Case-control	58.6 ± 20.1	213/78	291	127	164	43.64%	Sepsis3.0	GCS <15 OR CAM-ICU
Jin et al. (2022)	China	Cohort	75.3 ± 10.4	155/67	222	132	90	59.46%	Sepsis3.0	GCS <15 OR CAM-ICU
Yeunwoo et al. (2020)	Korea	Case-control	67.3 ± 15.0	95/80	175	107	68	61.14%	Sepsis1.0	CAM-ICU
Feng et al. (2021)	China	Cohort	53.0 ± 11.0	20/31	51	20	31	39.22%	Sepsis3.0	CAM-ICU
Duc et al. (2014)	Belgium	Case-control	65.0 ± 14.0	83/45	128	107	21	83.59%	Sepsis2.0	GCS <15 OR CAM-ICU
Feng et al. (2017)	China	Cohort	56.7 ± 15.0	107/68	175	74	101	42.29%	Sepsis1.0	CAM-ICU
Li et al.(2022)	China	Case-control	37.5 ± 4.5	18/23	41	21	20	51.22%	Diagnostic criteria for burn infection	Data recorded by medical staff

CAM-ICU, Confusion Assessment Method for the ICU; GCS, Glasgow Coma Scale. Sepsis 1.0, 2.0, 3.0, and diagnostic criteria for burn infection are all diagnostic methods for sepsis (11–13, 21).

When only 1 study provided data on the association between a potential risk factor and SAE, we reported the estimate extracted from the original article in the table (i.e., no meta-analysis was performed). When two or more studies provided data on the association between a potential risk factor and SAE, we calculated a meta-analyzed estimate of that possible association. When encountering studies that provided unadjusted and adjusted estimates, we preferred to use adjusted effect estimates because they represent effect estimates closer to the actual value (i.e., less biased). If forecasts from different overlapping cohorts were available, we used data from the report with the largest sample size to avoid duplicating data in the meta-analysis.

### Definitions and outcomes

The diagnostic criteria for sepsis are constantly being updated. There are also no uniform diagnostic criteria for sepsis-associated encephalopathy. In each study we included, the diagnosis of both conditions varied. If it does not violate general principles, we respect the diagnostic criteria for sepsis and sepsis-associated encephalopathy used by researchers in each study and directly extract patient information from the data published in each study. The primary outcome of this study was the risk factors of sepsis-associated encephalopathy.

### Pre-specified subgroup analysis and heterogeneity analysis

We will group the studies included based on the type of study design (case-control studies or cohort studies). Additionally, due to the lack of uniform diagnostic criteria for sepsis-associated encephalopathy, we will also group the studies based on whether the diagnosis in the included studies was made using objective scoring (GCS <15 or CAM-ICU) or based on the subjective evaluation of medical personnel. If heterogeneity analysis is required, we will determine the source of heterogeneity by employing a study-by-study exclusion approach for the included studies.

### Quality evaluation and certainty assessment

Two independent authors assessed the included studies’ methodological strength and risk of bias using the Newcastle–Ottawa Scale (NOS) (22). We used this assessment tool to evaluate the design quality of nonrandomized case-control and cohort studies. Scores were assigned based on selection criteria, comparability, and outcome (cohort studies) or exposure (case-control studies). The maximum score of 9 reflects the highest quality.

We used the Grades of Recommendations, Assessment, Development, and Evaluation (GRADE) evidence rating system to assess the credibility of the available evidence for the relevant factors associated with this meta-analysis (23, 24). We initially regarded observational studies as being of poor quality. Depending on the GRADE criteria, the level of credibility may be reduced (with five domains, including the risk of bias, inconsistency, circumstantial evidence, uncertainty, or publication bias) or increased (with three fields, including larger effect sizes, dose-response relationships, or confounders). We ultimately categorized the credibility of the evidence as high, moderate, low, or very low.

### Statistical analyses

We used Review Manager (version 5.3; Nordic Cochrane Centre, The Cochrane Collaboration, Copenhagen, Denmark) (25) to perform meta-analysis. Continuous variables were pooled using standard mean difference (SMD), and We calculated a 95% confidence interval (CI). In contrast, binary variables were pooled using odds ratio (OR), and a 95% confidence interval (CI) was calculated. A fixed effects model was used when statistical heterogeneity was low (*I*^2^ ≤ 50%). When statistical heterogeneity was high (*I*^2^ > 50%), meta-analysis was performed using a random-effects model, and sensitivity and subgroup analyses were performed to investigate potential sources of heterogeneity (26). *p* < 0.05 was considered statistically significant.

## Results

### Study process

**Figure 1:**
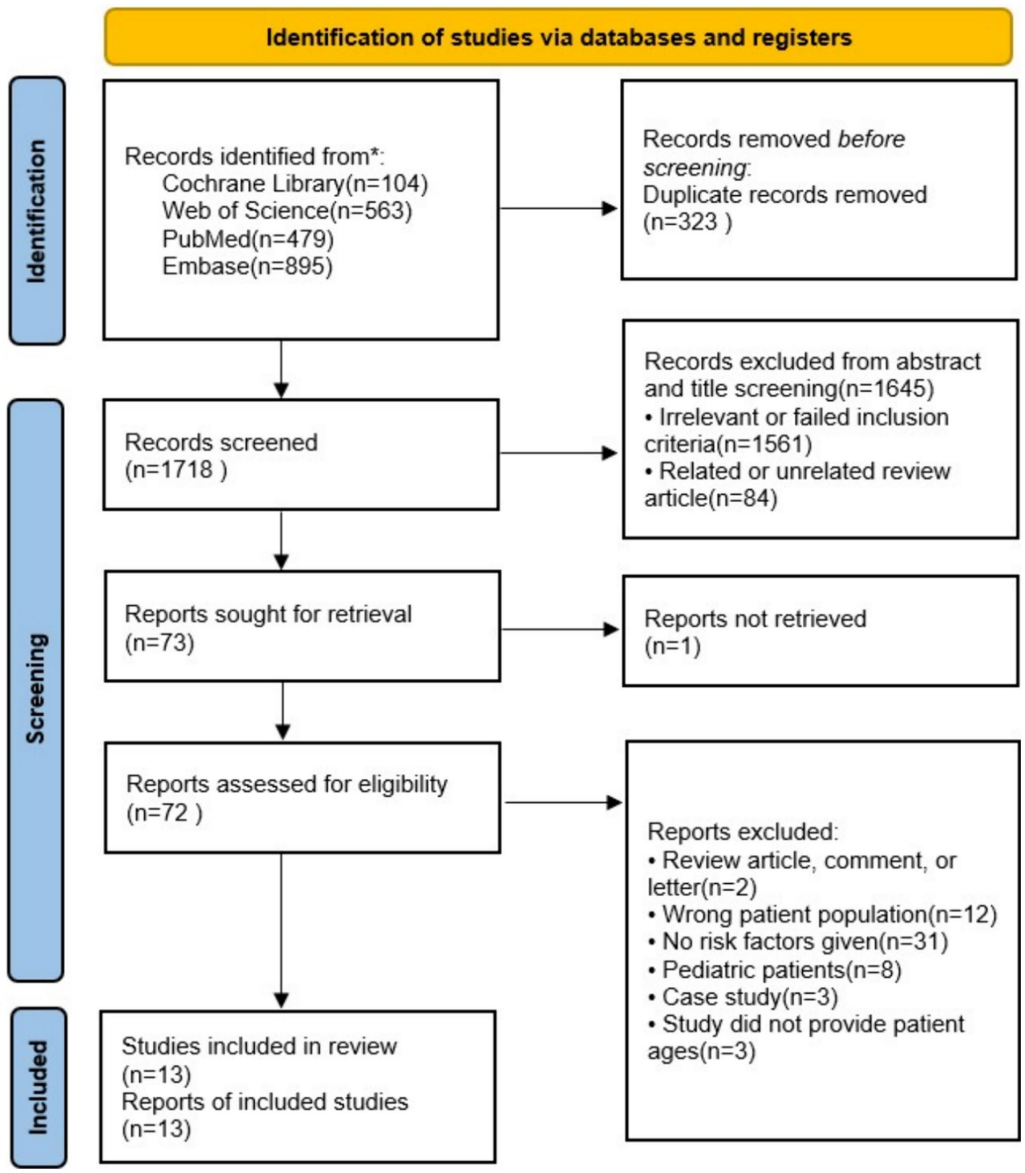
Flow diagram of the literature and selection in the meta-analysis.

A flow diagram for study selection is shown in Figure 1. Through database searching from inception to August 10, 2023, the date of our final search, we identified 2,041 original literature records. After the removal of duplicates, 1,718 records remained. Of these, 1,645 were excluded after screening titles and abstracts as they met our exclusion criteria. Excluding one article that could not be retrieved, we assessed the full texts of the remaining 72 articles. Ultimately, a total of 13 studies met the eligibility criteria for a full-text review (27–39).

### Study characteristics

Of the 13 studies included, there were six cohort studies and seven case-control studies. All these studies were published in English. The total sample size of these studies was 1906, with a cumulative case group of 864 and a control group of 1,042. The prevalence of SAE ranged from 17.67 to 83.59%. The studies were published between 2011 and 2023. The included studies were conducted in multiple countries, including China (*n* = 10), South Korea (*n* = 1), Finland (*n* = 1), and Belgium (*n* = 1). Thirteen studies were conducted in single-center settings. One study exclusively utilized the Glasgow Coma Scale (GCS) criteria for assessing SAE. Four studies solely employed the Confusion Assessment Method for the Intensive Care Unit (CAM-ICU) criteria for evaluating SAE. Three studies incorporated both GCS and CAM-ICU criteria for the assessment of SAE. Additionally, five studies assessed SAE through clinical records maintained by healthcare professionals (more details are seen in Table 1).

### Quality evaluation of included studies

According to the NOS scores, 12 were high-quality studies, and one was of moderate quality (Supplementary Table S3). When performing multivariate analyses, explanations of the factors that accounted for most of the lost points were made more evident.

### GRADE assessment of the certainty of the evidence

In our study, observational cohort studies were initially considered low quality. However, there was no high-quality evidence for any of the risk factors regarding deterministic assessment. Only APACHE II, SOFA, age, dependent activities, high care needs, low level of consciousness, and ALT showed moderate-quality evidence (Table 2). For all other risk factors, the certainty of evidence was categorized as low. The reduced quality of evidence for some risk factors was attributed to variations in study results and imprecision.

**Table 2:** Potential risk factors for SAE and its effect estimates.

Potential risk factor for SAE	Number of studies	Total sample	Effect estimates (95% CI), random-effects meta-analysis	Heterogeneity test		*p*-value	Effect size model	Certainty in the evidence using the GRADE approach
				*I*^2^	Chi^2^			
APACHE II	6	1,158	SMD 1.84 (0.63, 3.06)	98%	321.02	0.003	Random	Medium
SOFA	4	597	SMD 2.30 (0.35, 4.26)	98%	198.7	0.02	Random	Medium
GCS	1	232	SMD −0.94 (−1.28, −0.59)	NA	NA	NA	NA	Low
Age	3	688	SMD 1.24 (0.71, 1.78)	90%	20.28	<0.00001	Random	Medium
Hypertension	1	291	OR 1.82 (1.07, 3.10)	NA	NA	NA	NA	Low
COPD	1	222	OR 2.70 (1.34, 5.60)	NA	NA	NA	NA	Low
Dependent activity	1	175	OR 5.83 (2.85, 11.93)	NA	NA	NA	NA	Medium
High nursing needs	1	175	OR 3.75 (1.96, 7.17)	NA	NA	NA	NA	Medium
Low level of consciousness	1	175	OR 4.23 (1.90, 9.44)	NA	NA	NA	NA	Medium
Tachypnoea	1	175	OR 2.80 (1.49, 5.28)	NA	NA	NA	NA	Low
Gastrointestinal infections	1	291	OR 2.03 (1.14, 3.63)	NA	NA	NA	NA	Low
Detection rate of enterococcus	1	291	OR 2.30 (1.10, 4.80)	NA	NA	NA	NA	Low
Heart rate	1	232	SMD 0.52 (0.18, 0.86)	NA	NA	NA	NA	Low
PaO_2_	1	284	SMD −0.36 (−0.61, −0.12)	NA	NA	NA	NA	Low
ALT	1	284	SMD 0.37 (0.13, 0.61)	NA	NA	NA	NA	Medium
Blood lactate	1	232	SMD 0.43 (0.09, 0.77)	NA	NA	NA	NA	Low
Serum sodium	2	454	SMD −0.15 (−1.29, 0.99)	95%	21.47	0.8	Random	Low
Platelets	1	232	SMD −0.35 (−0.69, −0.01)	NA	NA	NA	NA	Low
Serum albumin	2	322	SMD −0.55 (−0.82, −0.28)	0%	0.78	<0.0001	Fixed	Low
PH	1	232	SMD −0.39 (−0.73, −0.05)	NA	NA	NA	NA	Low
Tau protein	2	150	SMD 1.52 (0.58, 2.47)	78%	4.48	0.002	Random	Low
MFI of CD86 in NKT	1	90	SMD-2.19 (−2.73,-1.65)	NA	NA	NA	NA	Low
CD4^+^	1	86	SMD −1.69 (−2.20, −1.19)	NA	NA	NA	NA	Low
IL-6	2	63	SMD 1.84 (0.24, 3.44)	85%	6.58	0.02	Random	Low
S100 β	1	22	SMD 1.65 (0.66, 2.65)	NA	NA	NA	NA	Low
THRR index <1.09	1	51	OR 5.78 (1.22, 27.26)	NA	NA	NA	NA	Low
Mean value for rSO_2_ <55%	1	51	OR 3.86 (1.02–14.55)	NA	NA	NA	NA	Low
Cortisol	2	169	SMD 3.67 (−3.01, 10.36)	98%	55.26	0.28	Random	Low
ACTH	1	41	SMD 2.73 (1.86, 3.60)	NA	NA	NA	NA	Low

CI, confidence interval; NA, not applicable; OR, odds ratio; RR, risk ratio; SMD, standard mean difference; PaO_2_, partial pressure of oxygen in arterial blood; ALT, alanine aminotransferase; APACHEII, Acute Physiology and Chronic Health Evaluation II; PH, potential of hydrogen; SOFA, Sequential Organ Failure Assessment; MFI of CD86 in NKT, Mean Fluorescence Intensity of CD86 in Natural Killer T cells; CD4^+^, cluster of differentiation 4; IL-6, interleukin-6; S100 β, S100 calcium-binding protein beta; COPD, chronic obstructive pulmonary disease; THRR, transient hyperemic response ratio; rSO_2_, regional cerebral oxygen saturation; ACTH, adrenocorticotropic hormone.

### Meta-analysis of risk factors for SAE

#### APACHE II

**Figure 2:**
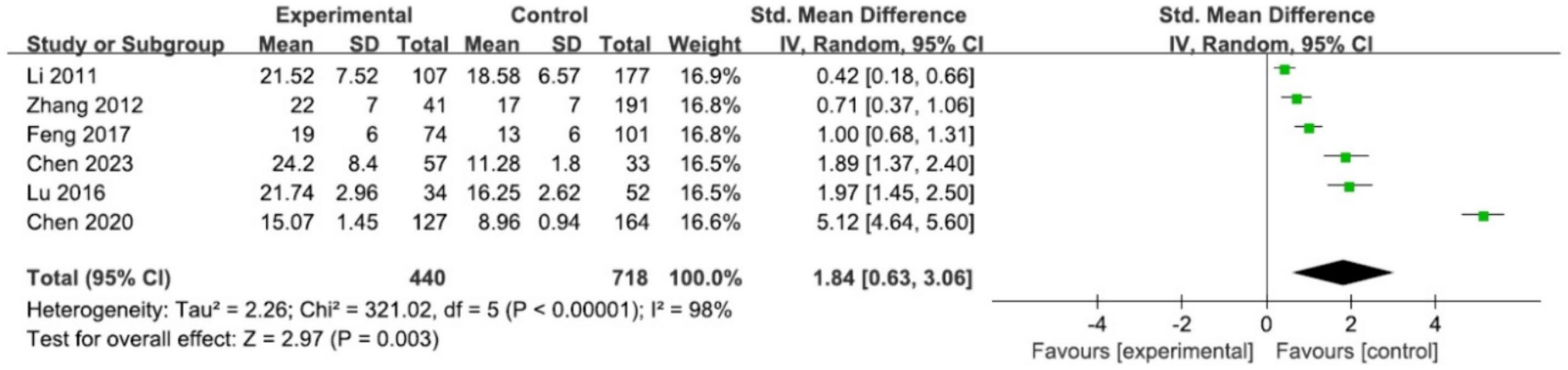
Forest plot of the studies in APACHEII.

We conducted a meta-analysis of data from 6 studies (27, 28, 30, 31, 33, 38) examining the relationship between APACHE II and SAE, with 440 cases in the SAE group and 718 cases in the non-SAE group. The results showed a statistically positive effect between the two {[SMD = 1.84, 95% CI (0.63, 3.06)], *p* = 0.003} (Table 2 and Figure 2). However, there was a high degree of heterogeneity between studies (*I*^2^ = 98%, *p* < 0.00001), and the source of the heterogeneity could not be determined despite a study-by-study exclusion maneuver. Subgroup analyses were performed based on differences in study design and diagnostic criteria for encephalopathy between studies (Supplementary Figures S1, S2).

#### SOFA

**Figure 3:**
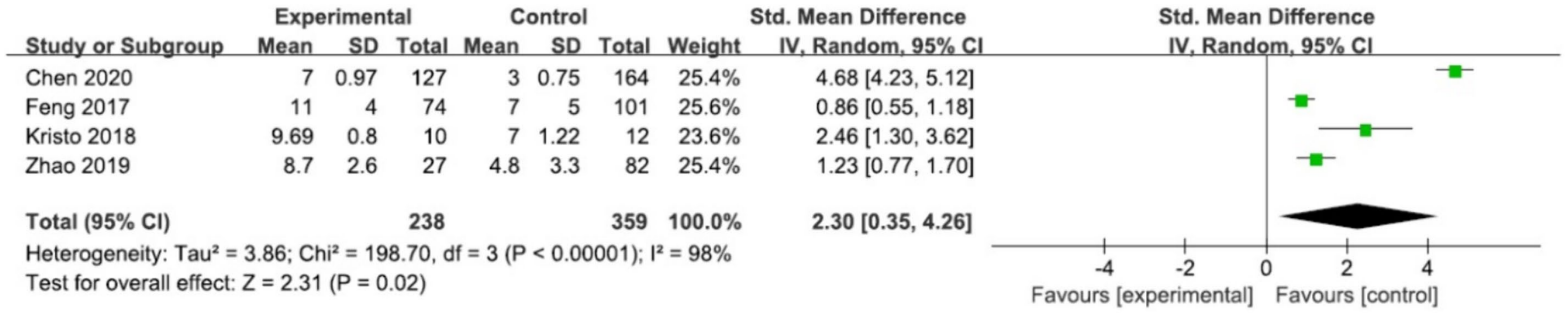
Forest plot of the studies in SOFA.

The effect of SOFA on SAE was examined in four studies (29, 32, 33, 38). There were 238 cases in the SAE group and 359 cases in the non-SAE group. The results demonstrated a statistically significant positive association between the two variables, as evidenced by a standard mean difference (SMD) of 2.3 with a 95% confidence interval (CI) ranging from 0.35 to 4.26 (*p* = 0.02) (Table 2 and Figure 3). Notably, the studies had substantial heterogeneity (*I*^2^ = 98%, *p* < 0.00001). Despite conducting a thorough exclusion analysis for each study, the root cause of this heterogeneity remained elusive. Consequently, subgroup analyses were undertaken to explore the potential effects of variations in study designs across the studies (Supplementary Figure S3).

#### Age

**Figure 4:**
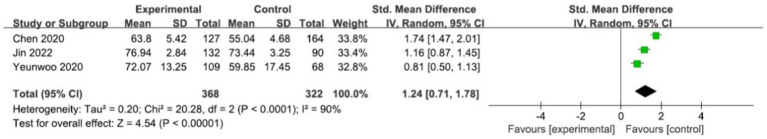
Forest plot of the studies in age.

In three studies, the impact of age on sepsis-associated encephalopathy (SAE) was examined, and a meta-analysis was conducted (33, 34, 38). The SAE group comprised 368 cases, while the non-SAE group included 322 cases. The results showed a statistically positive effect between the two {[SMD = 1.24, 95% CI (0.71, 1.78)], *p* < 0.00001} (Table 2 and Figure 4). However, there was a high degree of between-study heterogeneity (*I*^2^ = 90%, *p* < 0.0001), and despite a study-by-study exclusion maneuver, the source of the heterogeneity could not be determined. Subgroup analyses were then performed based on differences in study design between studies (Supplementary Figure S4).

#### Albumin and serum sodium

We conducted a meta-analysis of data from 2 studies (28, 30) examining the relationship between albumin and SAE, with 98 cases in the SAE group and 224 cases in the non-SAE group. The results showed a statistically negative effect between the two {[SMD = −0.55, 95% CI (−0.82, −0.28)], *p* < 0.0001} and low heterogeneity in the meta-analysis. In our meta-analysis (28, 34), sodium was not found to be a significant risk factor in the occurrence of SAE (*p* = 0.8) (Table 2 and Supplementary Figures S5, S6).

#### Tau protein, IL-6 and cortisol

The effect of tau protein on SAE was examined in 2 studies (29, 39). The results showed a statistically positive effect between the two {[SMD = 1.52, 95% CI (0.58, 2.47)], *p* = 0.002}, with high heterogeneity in the meta-analysis (*I*^2^ = 78%, *p* = 0.03). The effect of IL-6 on SAE was examined in 2 studies (32, 39). The results showed a statistically positive effect between the two {[SMD = 1.84, 95% CI (0.24, 3.44)], *p* = 0.02}, with high heterogeneity in the meta-analysis (*I*^2^ = 85%, *p* = 0.01). In our meta-analysis (37, 39), cortisol was not found to be a significant risk factor in SAE (*p* = 0.28) (Table 2 and Supplementary Figures S7–S9).

#### Other risk factors

In our study, multiple regression analysis identified 21 potential risk factors for developing encephalopathy in patients with sepsis. These factors include GCS, hypertension, COPD, dependent activity, high nursing needs, low level of consciousness, tachypnoea, gastrointestinal infections, detection rate of enterococcus, heart rate, PaO_2_, ALT, blood lactate, platelets, pH, MFI of CD86 in NKT cells, CD^4+^, S100 β, THRR index <1.09, mean value for rSO_2_ <55%, and ACTH. However, as these risk factors were only reported in a single study and insufficient data from multiple studies for a meta-analysis, they were omitted in our meta-analysis (more details seen in Table 2).

## Discussion

Six risk factors significantly associated with SAE were identified in this meta-analysis of 13 studies involving 1,906 patients hospitalized with sepsis. Among these, APACHE II, SOFA, age, tau protein, and IL-6 were found to be risk factors with significant effects (SMD: 1.24–2.30), and albumin was a risk factor with moderate effects (SMD: −0.55). However, for the risk factors identified in this meta-analysis, the certainty of evidence was low to moderate.

According to our results, the incidence of APACHE II and SOFA in SAE was statistically a risk factor for more significant effects, but considerable heterogeneity was seen between studies. We could not determine the heterogeneity source even after sensitivity analyses.

We performed subgroup analyses based on some subgroups that may affect heterogeneity, such as differences in study design and diagnostic criteria for encephalopathy. Despite these subgroups, there was still more significant heterogeneity in the within-group analyses of APACHE II and SOFA, suggesting that the significant inconsistency in the study results was not caused by differences in study design and diagnostic criteria for encephalopathy and that further exploration of the sources of heterogeneity is needed. Both APACHE II and SOFA scores are important indicators of the severity of a patient’s condition and have been widely used to evaluate critically ill patients (8, 40). Encephalopathy is seen as an indicator of a poor prognosis for patients suffering from sepsis; the severity of encephalopathy is closely related to the severity of the systemic disease and requires prompt and aggressive treatment. According to our study, both APACHE II and SOFA scores were risk factors for a more significant effect of SAE, with advantages in assessing the severity and prognosis of encephalopathy, consistent with previous reports (41). Compared to non-SAE patients, SAE patients are more severely ill, as evidenced by higher costs, prolonged hospitalization, and higher duration of mechanical ventilation (17–19). Also, patients with SAE had a higher hospital mortality rate, suggesting that once SAE occurs in septic patients, the mortality rate is significantly higher. In the studies included in our meta-analysis, the incidence of SAE in septic patients may be as high as 83.59% (37). The high incidence of SAE may explain why sepsis is still fatal today. It is important to note that GCS scores <15 were used to diagnose patients with SAE; however, GCS scores are a component of SOFA and APACHE II scores. Patients with higher SOFA and APACHE II scores were likelier to have SAE, which may have biased the conclusions (34).

In our study, age incidence in SAE was also statistically a risk factor for more significant effects, but considerable heterogeneity was seen between studies. Sensitivity analyses could not identify the source of the heterogeneity. Because only three studies were included, we did not perform subgroup analyses, and the small sample size made the results we obtained less reliable. There was a significant publication bias and other circumstances. Elderly patients are at higher risk of developing sepsis, and critically ill patients with underlying disease usually have a more rapid progression and a poorer prognosis. Especially if the underlying illness is hypertension COPD, these patients may be more likely to develop central nervous system complications (9, 42–44). In addition, hospitalization of elderly patients is often associated with an increase in the need for nursing care due to dependence on daily activities and poor self-care ability, which further aggravates their condition (45, 46). This finding is consistent with some of the SAE-associated risk factors identified in our META analysis, including Age, COPD, and others.

Laboratory parameters such as tau protein, IL-6, and albumin in our study, although risk factors for predicting a medium to significant effect for the occurrence of SAEs, were included in a limited number of studies with small sample sizes and were only supported by low-quality evidence. In our meta-analysis, we applied the GRADE system to ensure the scientific validity of our findings. Despite many studies initially rated as low quality, careful reassessment allowed for upgrades based on study design and execution. We recognize heterogeneity due to geographical and ethnic variations, which introduces some uncertainty in interpretation. Also, potential biases and unmeasured confounders in primary studies could affect the generalizability of our results. Our findings, while insightful, should be applied cautiously in clinical practice, integrating high-quality evidence, clinical experience, and patient values. They provide a basis for guidelines but require adaptation to local contexts. Future research should aim to enhance study design quality, increase sample sizes, and broaden demographic coverage to improve evidence quality and extend its applicability.

Further inflammation is needed in the future through more extensive studies supported by higher levels of evidence. However, IL-6 and albumin are conveniently available and cost-effective biomarkers during hospitalization because they can be calculated from standard peripheral blood tests without additional effort or expense. Monitoring changes in IL-6 and albumin levels can be a useful clinical tool for assessing the risk of SAE and developing appropriate medical interventions. This meta-analysis needed to have identified specific thresholds for predicting SAE due to the limited sample size and potential heterogeneity of baseline values in different studies. Future prospective studies are required to establish validated entries for predicting SAE. We confidently use IL-6 and Albumin as clinical tools for predicting SAE risk.

### Advantages and limitations

The main strength of this study is that it is the first meta-analysis of risk factors for SAE, where multiple factors affecting SAE were analyzed separately. However, like all studies, this study has some limitations. First, the diagnostic criteria for sepsis and sepsis-associated encephalopathy differed between studies. The diagnostic criteria for sepsis 1.0 have high sensitivity but low specificity, which may lead to overdiagnosis while missing some immunosuppressed patients. Sepsis 2.0 is a transitional criterion. However, the latest definition of sepsis 3.0 remains controversial. When a patient’s SOFA score changes by ≥2 points, the patient’s condition is exacerbated, caused by an infection that may adversely affect the early recognition and treatment of sepsis and lead to a delayed diagnosis of the disease. In addition, non-infectious conditions in critically ill patients may also lead to organ damage that can achieve a SOFA score of ≥2, leading to overdiagnosis. The diagnostic criteria for burn infection are expressly limited to burn patients (21). All four diagnostic criteria for sepsis are deficient. Among the currently used diagnostic criteria for SAE, GCS <15 and CAM-ICU are relatively objective diagnostic methods. At the same time, data recorded by medical staff is somewhat subjective; all three diagnostic procedures are based on consistent clinical symptoms of SAE. Second, only English-language databases were searched, possibly excluding relevant studies published in other languages. Third, only a few studies were included for certain factors such as tau protein, IL-6, and albumin, resulting in small sample sizes for these studies. Fourth, the exclusion of minors under the age of 18 limits the generalizability of the results. In addition, most of the studies were conducted in Asia and Europe, limiting the applicability of generalizing the results to other parts of the world.

Therefore, future research needs to address these limitations and provide a more comprehensive investigation of the risk factors associated with SAE. Efforts should include studies from different geographic regions and populations, consider a broader range of languages, and use standardized assessment methods. By overcoming these limitations, we can further advance our understanding of SAE and provide more substantial evidence for clinical practice.

## Data availability statement

The original contributions presented in the study are included in the article/Supplementary material, further inquiries can be directed to the corresponding author.

## Author contributions

ZZ: Writing – original draft. LG: Writing – review & editing. LJ: Writing – review & editing. HD: Writing – review & editing. LS: Writing – review & editing. HZ: Writing – review & editing.

## Figures and Tables

**Table:** 

SAE	Sepsis-related encephalopathy
BBB	Blood-brain barrier
ICU	Intensive care unit
GCS	Glasgow Coma Score
CAM-ICU	Confusion Assessment Method for the Intensive Care Unit
MeSH	Medical Subject Headings
NOS	Newcastle–Ottawa Scale
GRADE	Grading of Recommendations Assessment
SMD	Standard mean difference
CI	Confidence interval
OR	Odds ratio
NA	Not applicable
RR	Risk ratio
PaO_2_	Partial pressure of oxygen in arterial blood
ALT	Alanine aminotransferase
APACHEII	Acute Physiology and Chronic Health Evaluation II
PH	Potential of hydrogen
SOFA	Sequential Organ Failure Assessment
MFI of CD86 in NKT	Mean Fluorescence Intensity of CD86 in Natural Killer T cells
CD^4+^	Cluster of differentiation 4
IL-6	Interleukin-6
S100 β	S100 calcium-binding protein beta
COPD	Chronic obstructive pulmonary disease
THRR	Transient hyperemic response ratio
rSO_2_	Regional cerebral oxygen saturation
ACTH	Adrenocorticotropic hormone

## References

[1] Slooter AJC Otte WM Devlin JW Arora RC Bleck TP Claassen J . Updated nomenclature of delirium and acute encephalopathy: statement of ten societies. Intensive Care Med. (2020) 46:1020–2. doi: 10.1007/s00134-019-05907-4, PMID: 32055887 PMC7210231

[2] Wilson JX Young GB. Progress in clinical neurosciences: sepsis-associated encephalopathy: evolving concepts. Can J Neurol Sci. (2003) 30:98–105. doi: 10.1017/S031716710005335X, PMID: 12774948

[3] Iacobone E Bailly-Salin J Polito A Friedman D Stevens RD Sharshar T. Sepsis-associated encephalopathy and its differential diagnosis. Crit Care Med. (2009) 37:S331–6. doi: 10.1097/CCM.0b013e3181b6ed58 20046118

[4] Heming N Mazeraud A Verdonk F Bozza FA Chrétien F Sharshar T. Neuroanatomy of sepsis-associated encephalopathy. Crit Care. (2017) 21:65. doi: 10.1186/s13054-017-1643-z, PMID: 28320461 PMC5360026

[5] Sharshar T Annane D de la Grandmaison GL Brouland JP Hopkinson NS Françoise G. The neuropathology of septic shock. Brain Pathol. (2004) 14:21–33. doi: 10.1111/j.1750-3639.2004.tb00494.x 14997934 PMC8095740

[6] Bleck TP Smith MC Pierre-Louis SJ Jares JJ Murray J Hansen CA. Neurologic complications of critical medical illnesses. Crit Care Med. (1993) 21:98–103. doi: 10.1097/00003246-199301000-00019 8420739

[7] Czempik PF Pluta MP Krzych ŁJ. Sepsis-associated brain dysfunction: a review of current literature. Int J Environ Res Public Health. (2020) 17:5852. doi: 10.3390/ijerph17165852, PMID: 32806705 PMC7460246

[8] Eidelman LA Putterman D Putterman C Sprung CL. The spectrum of septic encephalopathy. Definitions, etiologies, and mortalities. JAMA. (1996) 275:470–3. doi: 10.1001/jama.1996.03530300054040 8627969

[9] Sonneville R de Montmollin E Poujade J Garrouste-Orgeas M Souweine B Darmon M . Potentially modifiable factors contributing to sepsis-associated encephalopathy. Intensive Care Med. (2017) 43:1075–84. doi: 10.1007/s00134-017-4807-z, PMID: 28466149

[10] Lu X Qin M Walline JH Gao Y Yu S Ge Z . Clinical phenotypes of SEPSIS-associated encephalopathy: a retrospective cohort study. Shock. (2023) 59:583–90. doi: 10.1097/SHK.0000000000002092, PMID: 36821412 PMC10082059

[11] Dellinger RP Levy MM Rhodes A Annane D Gerlach H Opal SM . Surviving sepsis campaign: international guidelines for management of severe sepsis and septic shock: 2012. Crit Care Med. (2013) 41:580–637. doi: 10.1097/CCM.0b013e31827e83af 23353941

[12] Dellinger RP Levy MM Carlet JM Bion J Parker MM Jaeschke R . Surviving sepsis campaign: international guidelines for management of severe sepsis and septic shock: 2008. Intensive Care Med. (2008) 34:17–60. doi: 10.1007/s00134-007-0934-2, PMID: 18058085 PMC2249616

[13] Rhodes A Evans LE Alhazzani W Levy MM Antonelli M Ferrer R . Surviving sepsis campaign: international guidelines for management of sepsis and septic shock: 2016. Intensive Care Med. (2017) 43:304–77. doi: 10.1007/s00134-017-4683-6, PMID: 28101605

[14] Young GB Bolton CF Austin TW Archibald YM Gonder J Wells GA. The encephalopathy associated with septic illness. Clin Invest Med. (1990) 13:297–304. PMID: 2078909

[15] Eggers V Schilling A Kox WJ Spies C. Septic encephalopathy. Diagnosis und therapy. Anaesthesist. (2003) 52:294–303. doi: 10.1007/s00101-003-0496-9, PMID: 12715131

[16] Chelazzi C Consales G De Gaudio A. Sepsis associated encephalopathy. Curr Anaesth Crit Care. (2008) 19:15–21. doi: 10.1016/j.cacc.2007.07.009

[17] Ren C Yao RQ Zhang H Feng YW Yao YM. Sepsis-associated encephalopathy: a vicious cycle of immunosuppression. J Neuroinflammation. (2020) 17:14. doi: 10.1186/s12974-020-1701-3, PMID: 31924221 PMC6953314

[18] Kikuchi DS Campos ACP Qu H Forrester SJ Pagano RL Lassègue B . Poldip2 mediates blood-brain barrier disruption in a model of sepsis-associated encephalopathy. J Neuroinflammation. (2019) 16:241. doi: 10.1186/s12974-019-1575-4, PMID: 31779628 PMC6883676

[19] Sonneville R Benghanem S Jeantin L de Montmollin E Doman M Gaudemer A . The spectrum of sepsis-associated encephalopathy: a clinical perspective. Crit Care. (2023) 27:386. doi: 10.1186/s13054-023-04655-8, PMID: 37798769 PMC10552444

[20] Moher D Liberati A Tetzlaff J Altman DG. Preferred reporting items for systematic reviews and meta-analyses: the PRISMA statement. PLoS Med. (2009) 6:e1000097. doi: 10.1371/journal.pmed.1000097, PMID: 19621072 PMC2707599

[21] Peng YZ Yuan ZQ Li XL Luo GX Wu J. Guidelines for the diagnosis and treatment of burn infection (2012 edition). Chin J Burns. (2012) 28:401–3. doi: 10.3760/cma.j.issn.1009-2587

[22] Wells GA Wells G Shea B Shea B O’Connell D Peterson J . (Eds). The Newcastle-Ottawa scale (NOS) for assessing the quality of nonrandomised studies in meta-analyses. (2014). Available at: https://www.ohri.ca/programs/clinical_epidemiology/oxford.asp

[23] Balshem H Helfand M Schünemann HJ Oxman AD Kunz R Brozek J . GRADE guidelines: 3. Rating the quality of evidence. J Clin Epidemiol. (2011) 64:401–6. doi: 10.1016/j.jclinepi.2010.07.015 21208779

[24] Foroutan F Guyatt G Zuk V Vandvik PO Alba AC Mustafa R . GRADE guidelines 28: use of GRADE for the assessment of evidence about prognostic factors: rating certainty in identification of groups of patients with different absolute risks. J Clin Epidemiol. (2020) 121:62–70. doi: 10.1016/j.jclinepi.2019.12.023, PMID: 31982539

[25] DerSimonian R Laird N. Meta-analysis in clinical trials revisited. Contemp Clin Trials. (2015) 45:139–45. doi: 10.1016/j.cct.2015.09.002, PMID: 26343745 PMC4639420

[26] Higgins JP Thompson SG Deeks JJ Altman DG. Measuring inconsistency in meta-analyses. BMJ. (2003) 327:557–60. doi: 10.1136/bmj.327.7414.557, PMID: 12958120 PMC192859

[27] Li J Li A Weng Y Zhang S Duan M. Risk factors for sepsis-associated encephalopathy. Neural Regen Res. (2011) 6:309–12. doi: 10.3969/j.issn.1673-5374

[28] Zhang LN Wang XT Ai YH Guo QL Huang L Liu ZY . Epidemiological features and risk factors of sepsis-associated encephalopathy in intensive care unit patients: 2008–2011. Chin Med J. (2012) 125:828–31. doi: 10.3760/cma.j.issn.0366-6999.2012.05.018 PMID: 22490582

[29] Zhao T Xia Y Wang D Pang L. Association between elevated serum tau protein level and sepsis-associated encephalopathy in patients with severe sepsis. Can J Infect Dis Med Microbiol. (2019) 2019:1–6. doi: 10.1155/2019/1876174 PMC6664571 31396296

[30] Chen SL Liu XY Huang JH Xian LH Li XS Wang KR . The expression of CD86 in CD3^+^ CD56^+^ NKT cells is associated with the occurrence and prognosis of sepsis-associated encephalopathy in sepsis patients: a prospective observational cohort study. Immunol Res. (2023) 71:929–40. doi: 10.1007/s12026-023-09405-0, PMID: 37405561

[31] Lu CX Qiu T Tong HS Liu ZF Su L Cheng B. Peripheral T-lymphocyte and natural killer cell population imbalance is associated with septic encephalopathy in patients with severe sepsis. Exp Ther Med. (2016) 11:1077–84. doi: 10.3892/etm.2016.3000, PMID: 26998040 PMC4774448

[32] Erikson K Ala-Kokko TI Koskenkari J Liisanantti JH Kamakura R Herzig KH . Elevated serum S-100β in patients with septic shock is associated with delirium. Acta Anaesthesiol Scand. (2019) 63:69–73. doi: 10.1111/aas.13228, PMID: 30079511

[33] Chen J Shi X Diao M Jin G Zhu Y Hu W . A retrospective study of sepsis-associated encephalopathy: epidemiology, clinical features and adverse outcomes. BMC Emerg Med. (2020) 20:77. doi: 10.1186/s12873-020-00374-3, PMID: 33023479 PMC7539509

[34] Jin G Wang S Chen J Hu W Zhu Y Xi S. Identification of sepsis-associated encephalopathy risk factors in elderly patients: a retrospective observational cohort study. Turk J Med Sci. (2022) 52:1513–22. doi: 10.55730/1300-0144.5491 36422495 PMC10395672

[35] Kim Y Jin Y Jin T Lee SM. Risk factors and outcomes of sepsis-associated delirium in intensive care unit patients: a secondary data analysis. Intensive Crit Care Nurs. (2020) 59:102844. doi: 10.1016/j.iccn.2020.102844, PMID: 32253122

[36] Feng Q Ai M Huang L Peng Q Ai Y Zhang L. Relationship between cerebral hemodynamics, tissue oxygen saturation, and delirium in patients with septic shock: a pilot observational cohort study. Front Med. (2021) 8:641104. doi: 10.3389/fmed.2021.641104, PMID: 34901041 PMC8660998

[37] Nguyen DN Huyghens L Zhang H Schiettecatte J Smitz J Vincent JL. Cortisol is an associated-risk factor of brain dysfunction in patients with severe sepsis and septic shock. Biomed Res Int. (2014) 2014:712742:1–7. doi: 10.1155/2014/712742 24883321 PMC4022165

[38] Feng Q Ai YH Gong H Wu L Ai ML Deng SY . Characterization of sepsis and sepsis-associated encephalopathy. J Intensive Care Med. (2019) 34:938–45. doi: 10.1177/0885066617719750 28718340

[39] Li XL Xie JF Ye XY Li Y Li YG Feng K . Value of cerebral hypoxic-ischemic injury markers in the early diagnosis of sepsis associated encephalopathy in burn patients with sepsis. Zhonghua Shao Shang Yu Chuang Mian Xiu Fu Za Zhi. (2022) 38:21–8. doi: 10.3760/cma.j.cn501120-20211006-00346 35152685 PMC11705286

[40] Sprung CL Peduzzi PN Shatney CH Schein RM Wilson MF Sheagren JN . Impact of encephalopathy on mortality in the sepsis syndrome. The Veterans Administration Systemic Sepsis Cooperative Study Group. Crit Care Med. (1990) 18:801–6. doi: 10.1097/00003246-199008000-00001 2379391

[41] Taylor SL Morgan DL Denson KD Lane MM Pennington LR. A comparison of the Ranson, Glasgow, and APACHE II scoring systems to a multiple organ system score in predicting patient outcome in pancreatitis. Am J Surg. (2005) 189:219–22. doi: 10.1016/j.amjsurg.2004.11.010, PMID: 15720995

[42] Hajjar I Keown M Frost B. Antihypertensive agents for aging patients who are at risk for cognitive dysfunction. Curr Hypertens Rep. (2005) 7:466–73. doi: 10.1007/s11906-005-0043-y 16386204

[43] Sonneville R Vanhorebeek I den Hertog HM Chrétien F Annane D Sharshar T . Critical illness-induced dysglycemia and the brain. Intensive Care Med. (2015) 41:192–202. doi: 10.1007/s00134-014-3577-0 25465908

[44] Siew ED Fissell WH Tripp CM Blume JD Wilson MD Clark AJ . Acute kidney injury as a risk factor for delirium and coma during critical illness. Am J Respir Crit Care Med. (2017) 195:1597–607. doi: 10.1164/rccm.201603-0476OC, PMID: 27854517 PMC5476907

[45] Pandharipande PP Girard TD Jackson JC Morandi A Thompson JL Pun BT . Long-term cognitive impairment after critical illness. N Engl J Med. (2013) 369:1306–16. doi: 10.1056/NEJMoa1301372, PMID: 24088092 PMC3922401

[46] Brummel NE Jackson JC Pandharipande PP Thompson JL Shintani AK Dittus RS . Delirium in the ICU and subsequent long-term disability among survivors of mechanical ventilation. Crit Care Med. (2014) 42:369–77. doi: 10.1097/CCM.0b013e3182a645bd, PMID: 24158172 PMC3947028

