# Supplementary material for: Factors contributing to sepsis-associated encephalopathy: a comprehensive systematic review and meta-analysis
Source: Front Med (Lausanne). 2024 May 21;11:1379019. doi: 10.3389/fmed.2024.1379019 (PMC11148246; doi:10.3389/fmed.2024.1379019)

Supplementary Material

Supplementary Table S3.Quality assessment of the 13 studies using the Newcastle–Ottawa Scale.

| Study | NOS |  |  |  |
| --- | --- | --- | --- | --- |
|  | Selection | Comparability | Outcome | Total score |
| Li et al.(2011) | 3 | 2 | 3 | 8 |
| Zhang et al.(2012) | 4 | 2 | 2 | 8 |
| Zhao et al.(2019) | 3 | 2 | 3 | 8 |
| Chen et al.(2023) | 3 | 2 | 3 | 8 |
| Lu et al.(2016) | 3 | 2 | 3 | 8 |
| Kristo et al.(2018) | 3 | 2 | 2 | 7 |
| Chen et al.(2020) | 3 | 2 | 3 | 8 |
| Jin et al.(2022) | 3 | 2 | 3 | 8 |
| Yeunwoo et al.(2020) | 4 | 2 | 2 | 8 |
| Feng et al.(2021) | 3 | 2 | 2 | 7 |
| Duc et al.(2014) | 4 | 2 | 2 | 7 |
| Feng et al.(2017) | 3 | 2 | 2 | 7 |
| Li et al.(2022) | 3 | 1 | 2 | 6 |

Supplementary Figure S1. Subgroup analysis of APACHE II based on study design.


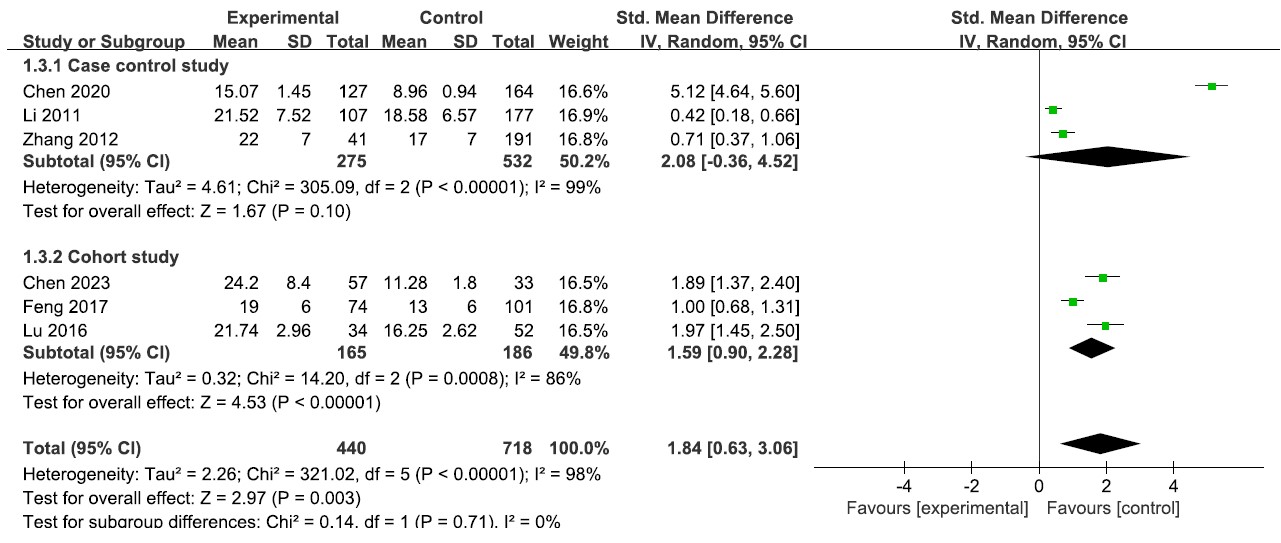


Supplementary Figure S2. Subgroup analysis of APACHE II based on diagnostic criteria for encephalopathy.


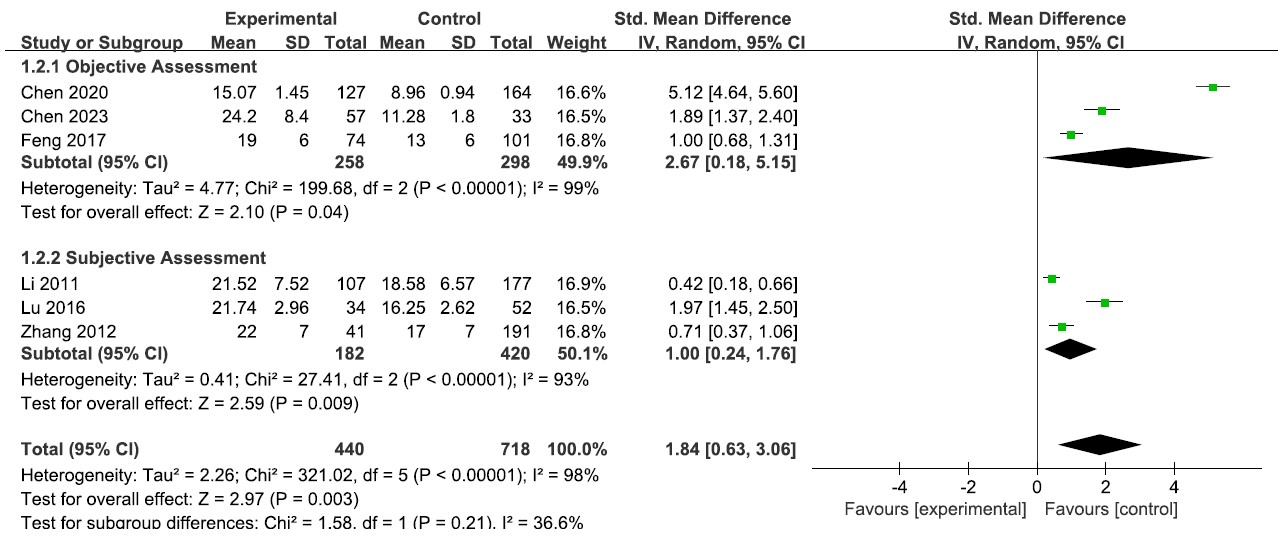


Supplementary Figure S3. Subgroup analysis of SOFA based on study design.


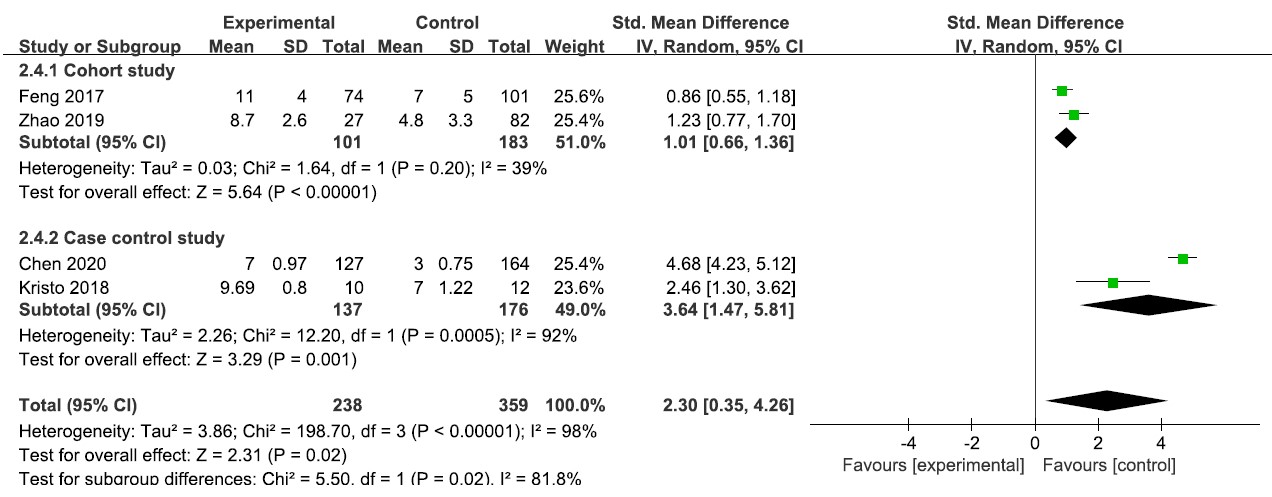


Supplementary Figure S4. Subgroup analysis of Age based on study design.


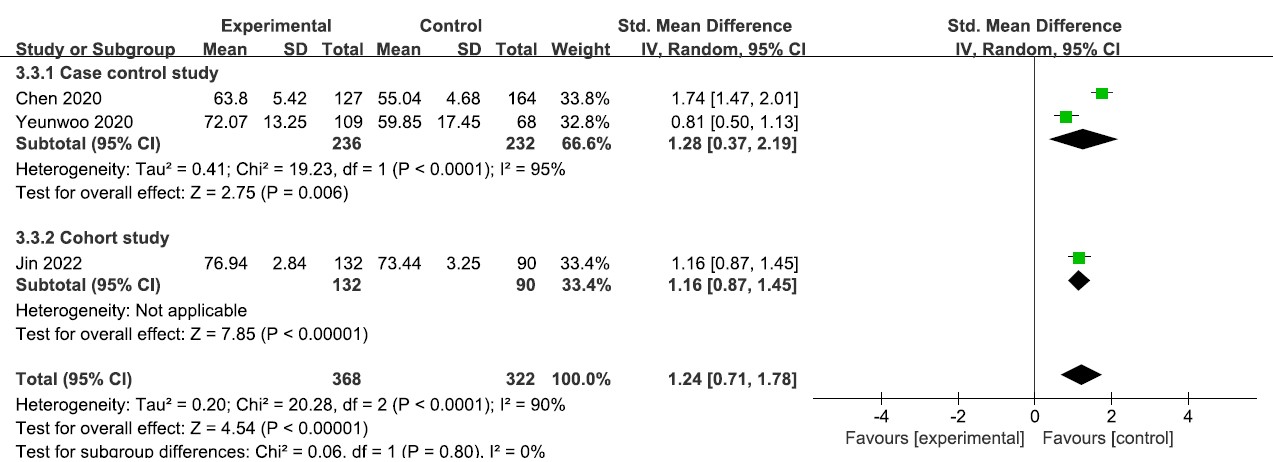


Supplementary Figure S5. Forest plot of the studies in Albumin.


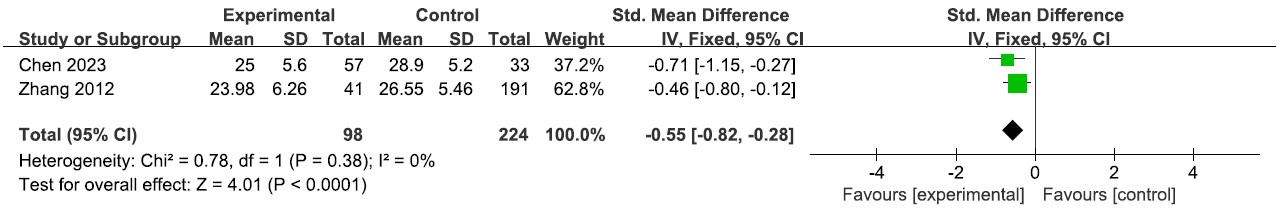


Supplementary Figure S6. Forest plot of the studies in Serum sodium.


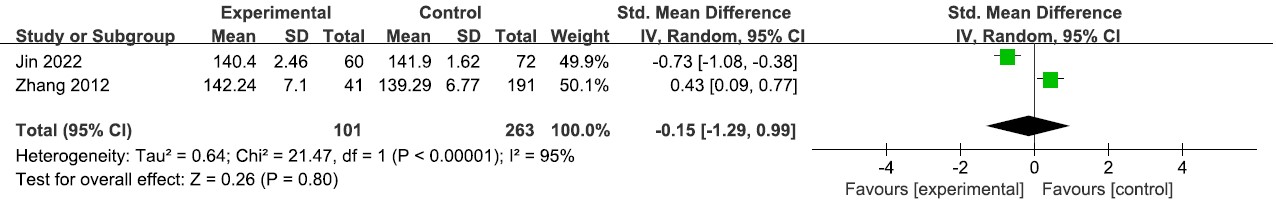


Supplementary Figure S7. Forest plot of the studies in Tau protein.


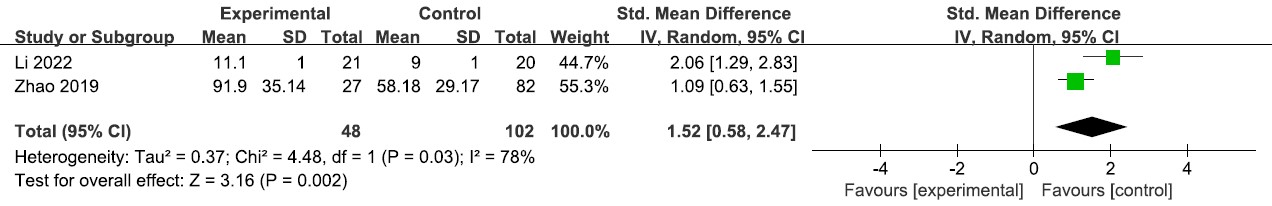


Supplementary Figure S8. Forest plot of the studies in IL-6.


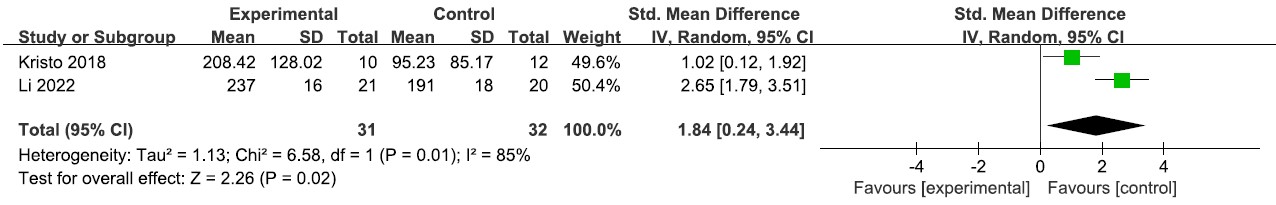


Supplementary Figure S9. Forest plot of the studies in Cortisol.


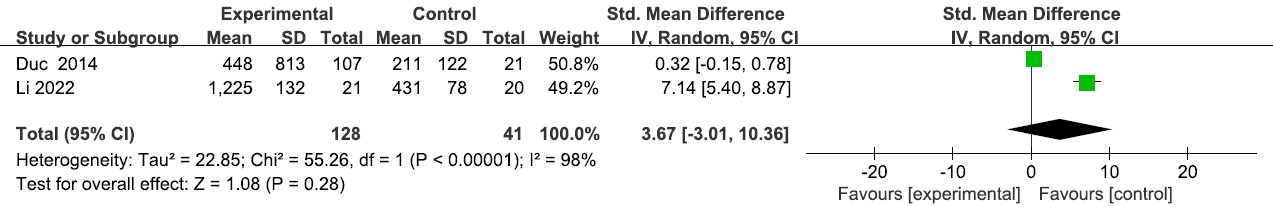

Supplement: Supplementary file 1 [file Data_Sheet_1.docx]
